# Pre‐screening of sleep‐disordered breathing after stroke: A systematic review

**DOI:** 10.1002/brb3.1146

**Published:** 2018-10-29

**Authors:** Mari Takala, Juha Puustinen, Esa Rauhala, Anu Holm

**Affiliations:** ^1^ Unit of Clinical Neurophysiology Satakunta Central Hospital Pori Finland; ^2^ Unit of Neurology Satakunta Central Hospital Pori Finland; ^3^ Division of Pharmacology and Pharmacotherapy University of Helsinki Helsinki Finland; ^4^ Social Security Centre of Pori Pori Finland; ^5^ Faculty of Health and Welfare Satakunta University of Applied Sciences Pori Finland

**Keywords:** pre‐screening, sleep‐disordered sleeping, stroke, systematic review

## Abstract

**Objectives:**

Sleep‐Disordered Breathing (SDB) is frequent in stroke patients. Polysomnography (PSG) and cardiorespiratory polygraphy are used to confirm SDB, but the need for PSG exceeds the available resources for systematic testing. Therefore, a simple and robust pre‐screening instrument is necessary to identify the patients with an urgent need for a targeted PSG. The aim of this systematic review was to identify and evaluate the available methods to pre‐screen stroke patients possibly suffering from SDB.

**Materials and Methods:**

Eleven studies out of 3,561 studies met the inclusion criteria. The selected studies assessed the efficiency of seven instruments based on the data acquired clinically or by inquiries (Berlin Questionnaire, Epworth Sleepiness Scale, SOS, Modified Sleep Apnea Scale of the Sleep Disorders Questionnaire, STOP‐BANG, Four‐variable Screening Tool and Multivariate Apnea Index) and three physiological measures (capnography, nocturia, nocturnal oximetry). The instruments were used to predict SDB in patients after acute or subacute stroke. Either PSG or cardiorespiratory polygraphy was used as a standard to measure SDB.

**Results:**

No independent studies using the same questionnaires, methods or criteria were published reducing generalizability. Overall, the questionnaires were quite sensitive in finding SDB but not highly specific in identifying the non‐affected. The physiological measures (capnography) indicated promising results in predicting SDB, but capnography is not an ideal pre‐screening instrument as it requires a specialist to interpret the results.

**Conclusions:**

The results of pre‐screening of SDB in acute and subacute stroke patients are promising but inconsistent. The current pre‐screening methods cannot readily be referred to clinicians in neurologic departments. Thus, it is necessary to conduct more research on developing novel pre‐screening methods for detecting SDB after stroke.

## 1 INTRODUCTION

Cerebrovascular diseases are a significant cause of disability and the second most common cause of death constituting approximately 10% of all deaths worldwide (Lopez, Mathers, Ezzati, Jamison & Murray, 2006). Several studies have addressed the increased prevalence of Sleep‐Disordered Breathing (SDB) in cerebrovascular accident (hereafter stroke) patients with an estimated prevalence of up to 50%–70% (Bassetti, Aldrich, Chervin & Quint, 1996; Cam, Gao, Imbach, Hodor & Bassetti, 2013; Gao, Cam, Jaeger, Zunzunegui & Sarnthein, 2010; Hermann & Bassetti, 2009; Martínez‐García, Soler‐Cataluña, Ejarque‐Martínez, Soriano & Román‐Sánchez, 2009; Sahlin, Sandberg, Gustafson, Bucht & Carlberg, 2008; Yaggi, Concato, Kernan, Lichtman & Brass, 2005), whereas in general SDB affects around 5%–10% of adult population (Lopez et al., 2006). SDB is evaluated to be an equivalent public health problem with smoking in society (Phillipson, 1993).

An increasing number of researches address the need for systematic SDB screenings after stroke. Untreated sleep disorders can increase the risk for recurrent strokes (Yaggi et al., 2005), whereas treatment of SDB with continuous positive airway pressure may reduce mortality after stroke (Martínez‐García et al., 2009). Moreover, adherence to sleep apnea treatment reduces the mortality rate as compared to untreated patients (Ou, Chen, Zhuo, Tian & He, 2015). However, some disagreement remains (McEvoy, Antic, Heeley, Luo & Ou, 2016). Sleep apnea is listed as a risk factor as well as a consequence of stroke in the European guidelines for cerebrovascular disease (ESO, 2008). Therefore, recognition and treatment of SDB after stroke constitute an important part of the secondary prevention and rehabilitation process. Early identification and treatment of SDB could enhance rehabilitation and decrease the patients’ time in hospital as well as increase the quality of life.

A polysomnography (PSG) or cardiorespiratory polygraphy are standard methods needed to diagnostically assess the severity of SDB. They are used to measure the Apnea‐Hypopnea Index (AHI) indicating the mean number of apnea or hypopnea events per hour. Unfortunately, there are more stroke patients than resources available for systematic PSG testing. SDB pre‐screening after stroke can also be considered an action in the prevention of recurrent stroke which could be beneficial in reducing disability and mortality in the long run. Thus, a simple targeted SDB pre‐screening method, which can potentially identify patients who should undergo more formal PSG, is needed. Early identification and treatment can boost rehabilitation, reduce time spent in hospitals and prevent recurrent strokes. As a result, ideally the economic burden that public health care poses on society could be reduced and great financial savings made.

Identifying predictive signs of SDB could help in finding the patients who benefit from the administration of PSG. Ideal SDB pre‐screening should be simple and fast for the medical or nursing personnel to administer, and it should not require a specialist's interpretation. The method should be sensitive in finding the patients at risk. Specificity could then be confirmed with more thorough PSG testing. The present systematic review aims to assess and evaluate current literature on existing SDB pre‐screening methods after acute or subacute cerebrovascular stroke and the predictive power of such methods.

## 2 METHODS

**Figure 1:**
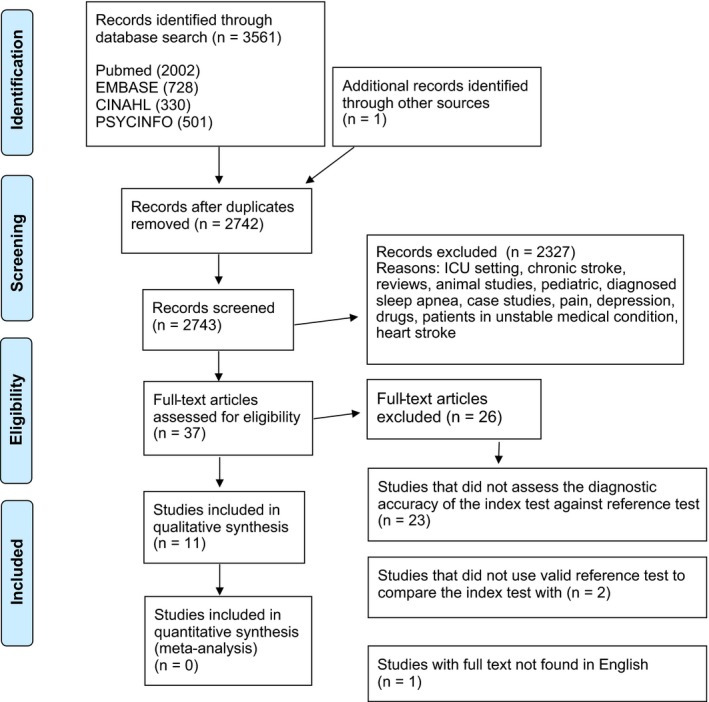
Flow chart of the articles reviewed, excluded and analyzed for the present systematic review

A systematic review was conducted according to the PRISMA (Preferred Reporting Items for Systematic Reviews and Meta‐Analyses) guideline (PRISMA guideline). This systematic approach was selected because the focus was solely on experimental articles, and the aim was to include all available experimental evidence. The literature search was carried out with the following electronic databases PubMed (MEDLINE), EMBASE, CINAHL and PsycINFO (from the earliest to October 28th 2016) (Figure 1). The search strategy was created by the present research group (MT, JP, ER, AH) assisted by an information specialist.

The search terms consisted of the following index terms “sleep,” “screening” and “stroke,” and additional search terms related to these index words such as “assessment,” “evaluation,” “questionnaire,” “monitor,” “measure,” “quality,” “scale,” “polygraph,” “polysomnography,” “actigraph,” “actometer,” “stroke,” “cerebral infarct,” “cerebral hemorrhage or cerebral haemorrhage,” “TIA or transient ischemic attack” or “cerebral ischemia” (Figure 1). No additional filters were included. In addition, the reference lists of the selected articles were checked.

The inclusion criteria for the studies consisted of the following:

- The study was conducted on acute (within an initial stay at the hospital due to the first onset of stroke) or subacute (within 1 year after stroke) cerebrovascular stroke patients (transient ischemic attack, cerebral infarct or intracerebral hemorrhage);
- The study used a pre‐screening method to predict SDB with calculated sensitivity and specificity;
- The study used either PSG or cardiorespiratory polygraphy as a standard to measure AHI and to compare the index test with; and
- The full text of the study was written in English.

First, the abstracts of the articles were reviewed by two researchers (MT, AH) blindly and independent of each other. The other members of the research group (JP, ER) were consulted if any disagreements occurred. The final decision required all members’ full agreement. Second, the data were extracted by two reviewers (MT, AH) in collaboration. The final extraction included the entire research group (MT, AH, JP, ER). Study characteristics, sensitivities and specificities and negative and positive predictive values (NPV, PPV) were collected from each paper as thoroughly as they were reported. Only reported results from each paper were included excluding any data requiring extrapolations or derivations from graphs or tables. The results from the studies with acute and subacute strokes were pooled together in our analysis. Studies with chronic phases were excluded.

The internal and external validities were assessed for each article according to Cochrane Methods Group on Screening and Diagnostic Tests guideline (Reitsma et al., 2009). Internal validity consisted of the following factors: valid standard test, definition of AHI by a standard test (full polysomnography or cardiorespiratory polygraphy), blind execution of tests, verification bias, and independent analysis of standard and index tests. External validity consisted of the following factors: disease spectrum, background information, cutoff values, missing data, index test completion, and the method for subject selection.

## 3 RESULTS

### 3.1 Included and excluded studies

The search strategy resulted in 3,561 research articles. The reasons for exclusion included review articles, animal studies, pediatric studies, case studies, studies conducted in patients with depression, pain, existing known or diagnosed sleep apnea, myocardial stroke, unstable medical condition or chronic stroke (more than one year after stroke onset), and drug studies (Figure 1). Finally, full texts were drawn for 37 studies of which 11 (Aaronson, Bezeij, Aardweg, Bennekom & Hofman, 2012; Bassetti, Aldrich, Chervin & Quint, 2006; Boulos, Wan, Im, Elias & Frankul, 2015; Broadley, Jørgesen, Cheek, Salonikis & Taylor, 2007; Camilo, Sander & Eckeli, 2014; Chen, Hsu, Pei, Yu & Chen, 2011; Dziewas, Hopmann, Humpert, Böntert & Dittrich, 2005; Elkholy, Amer, Nada, Nada & Labib, 2012; Katzan, Thompson, Uchino & Foldvary‐Schaefer, 2016; Kotzian, Stanek, Pinter, Grossmann & Saletu, 2012; Srijithesh, Shukla, Srivastav, Goyal & Singh, 2011) fulfilled the pre‐set inclusion criteria completely, and they were selected for the final analysis (Figure 1; Table 1). The rest of the 37 full texts, that is, 26 studies^S1–S26^, which passed the initial screening on the basis of the abstract but were eliminated in the final stage, when full texts were evaluated, are overviewed in the Appendix S1. They^S1–S26^ did not report diagnostic accuracy, had an invalid standard test, only assessed the prevalence of SDB or were not written in English. The studies were published between January 2005 and May 2016.

**Table 1:** The background information of the selected studies

Authors, publication year	Time from stroke (d)	Study setting	Screening method	No. of patients (apnea tested/total)	Mean age (*SD*)	Male (%)	BMI (*SD*)	AHI prevalence (%)	Stroke severity	Stroke subtype	AHI cutoff for SDB
Questionnaires or prediction model											
Boulos et al. (2015)	<180	N or O	4‐variable STOP‐BAG Berlin SOS	69	68.3 (14.2)	47.8	28.2 (6.3)	AHI ≥ 10 46.4% AHI ≥ 15 30.4%	NIHSS (Md) 1.5	Ischemic 46 Hemorrhage 7 TIA 16	AHI ≥ 10, AHI ≥ 15
Katzan et al. (2016)	Md = 235	S	STOP STOP‐BANG STOP‐BAG STOP‐BANG_2_ STOP‐BAG_2_ STOP‐BAG_2+_	208	55.5 (14.1)	51.0	30.9	AHI ≥ 10 61.0%		Ischemic stroke 99 hemorrhage 12 Other 97	AHI ≥ 10
Camilo et al. (2014)	30.3	E	BQ, ESS, SOS‐score	39	62.3 ± 12.2	64.1	26.7 (4.7)	AH I ≥ 10 77%	NIHSS (Md) 11	TOAST (8/18/6/0/7)	AHI ≥ 10
Elkholy et al. (2012)	<2	N	BQ	30/50	50.7 ± 14.9	60.0	50% BMI > 30	AHI > 10 56%		Ischemic 15, TIA 15	AHI ≥ 5, AHI > 15, AHI > 30
Kotzian et al. (2012)	1	N	BQ	68/515	63 ± 11	71.0	29 (5)	AHI ≥ 15 55%	BI 78 (27)	Stroke 68	AHI ≥15
Srijithesh et al. (2011)	<2	C	BQ, ESS, combination	39/121	56.5			AHI ≥ 5 56%	GCS 14	Infarct 76 Intracerebral hemorrhage 45	AHI ≥ 5, AHI ≥ 10
Broadley et al. (2007)		N	MAP index	55	71	58.0	26.8 (3.9)	AHI ≥ 10 58%	BI (mean) 53	Ischemic stroke 49 Hemorrhage 6	AHI ≥ 10
Bassetti et al. (2006)	<2	N	P‐SA	36/59	61 ± 11	61.0	27.4 (5)	AHI ≥ 10 69%	SSS (mean) 37 (15)	TIA 36 Stroke 63	AHI ≥ 10, AHI ≥ 20
Physiological measures											
Aaronson et al. (2012)	7–28	R	Nocturnal oximetry	67	55.6 ± 10.3	62.5	SAS 27.2 (4.6) No‐SAS 24.2 (4.6)	AHI ≥ 15 46%	16 moderately disabled 26 moderately severely disabled 14 severely disabled	Ischemic 41 Hemorrhage 11 Both 4	AHI ≥ 15
Chen et al. (2011)	240	R	Nocturia	74	AHI<30: 62.6 (11.5) AHI ≥ 30: 69.6 (9.9)	70.4	AHI < 30 23.9 (3.4) AHI≥30 25.0 (3.7)	AHI ≥ 30 55%	BI (mean) (score <80) 51/65	Ischemic stroke 65	AHI > 30
Dziewas et al. (2005)	<30	N	Capnograph	27	65.4 ± 14.1	70.4	25.7 (4)	AHI > 10 56%	NIHSS 10 (7)	Ischemic 27 (100)	AHI > 5, 10, 20

A, acute; AHI, Apnea‐Hypopnea Index (#/hr); BI, Barthel Index; BQ, Berlin Questionnaire; C, care referral; E, emergency unit; ESS, Epworth Sleepiness Scale; GCS, Glascow coma scale/score; MAP, Multivariate Apnea; N, neurology unit; NIHSS, National Institutes of Health Stroke Scale; O, outpatient clinic; P‐SA, Probability of Sleep Apnea Scale of the Sleep Disorders Questionnaire; R, neurology rehabilitation; S, sleep clinic; S, subacute; SSS, Scandinavian Stroke Scale; TIA, transient ischemic attack; TOAST, Trial of Org 10172 in Acute Treatment (large artery atherosclerosis/cardioembolism/small‐vessel occlusion/other causes/undetermined etiology).

### 3.2 Study characteristics

The study characteristics are shown in detail in Table 1. The studies were conducted in the following countries: Brazil, Egypt, Austria, India, USA, the Netherlands, Taiwan, Canada, Germany, the United States of America and Australia (Aaronson et al., 2012; Bassetti et al., 2006; Boulos et al., 2015; Broadley et al., 2007; Camilo et al., 2014; Chen et al., 2011; Dziewas et al., 2005; Elkholy et al., 2012; Katzan et al., 2016; Kotzian et al., 2012; Srijithesh et al., 2011). The majority of the studies were conducted in hospital settings: six in neurology units (Boulos et al., 2015; Broadley et al., 2007; Dziewas et al., 2005; Elkholy et al., 2012; Kotzian et al., 2012), one in an emergency unit (Camilo et al., 2014), two in rehabilitation units (Aaronson et al., 2012; Chen et al., 2011), one in a care referral teaching unit (Srijithesh et al., 2011), and one in a sleep clinic (Katzan et al., 2016). Apnea and hypopneas were frequent in the studied patients as the reported prevalence ranged from 46% to 77%. The studies included in the present systematic review used different cutoff values for SDB diagnosing as the ranking ranged from mild to severe (AHI ≥ 5 to AHI ≥ 30) (Aaronson et al., 2012; Bassetti et al., 2006; Boulos et al., 2015; Broadley et al., 2007; Camilo et al., 2014; Chen et al., 2011; Dziewas et al., 2005; Elkholy et al., 2012; Katzan et al., 2016; Kotzian et al., 2012; Srijithesh et al., 2011). The stroke severities were evaluated with the National Institutes of Health Stroke Scale (NIHSS) (Boulos et al., 2015; Dziewas et al., 2005), Barthel Index (BI) (Broadley et al., 2007; Chen et al., 2011; Kotzian et al., 2012), Scandinavian Stroke Scale (SSS) (Bassetti et al., 2006), Glasgow Coma Scale (GCS)(Srijithesh et al., 2011). One study (Broadley et al., 2007) merely described the severity of the strokes, and two studies (Elkholy et al., 2012; Katzan et al., 2016) failed to report information regarding stroke severity. Stroke etiologies were described in nine (Aaronson et al., 2012; Bassetti et al., 2006; Boulos et al., 2015; Broadley et al., 2007; Chen et al., 2011; Dziewas et al., 2005; Elkholy et al., 2012; Katzan et al., 2016; Srijithesh et al., 2011) out of eleven studies. In total, ischemic infarct was the reported etiology in 549 patients, TIA in 67, and hemorrhage in 81 patients. Additionally, one study (Camilo et al., 2014) reported the etiology according to the Trial of Org 10172 in Acute Treatment (TOAST) with eight cases of large artery atherosclerosis, 18 cardioembolisms, 6 small‐vessel occlusions and 7 with undetermined etiologies.

### 3.3 Subject characteristics

The selected 11 articles (Aaronson et al., 2012; Bassetti et al., 2006; Boulos et al., 2015; Broadley et al., 2007; Camilo et al., 2014; Chen et al., 2011; Dziewas et al., 2005; Elkholy et al., 2012; Katzan et al., 2016; Kotzian et al., 2012; Srijithesh et al., 2011) included 1,284 participants with 712 stroke patients tested for sleep apnea. In total, the sample size varied from 27 to 515 subjects. The mean age of the SDB tested participants ranged from 50 to 71 years, and male ratios from 47.8% to 70.0%. The mean body mass index (BMI) varied between 26.0 and 30.9 kg/m². The patients were tested for SDB in acute (within their initial stay at the hospital) or subacute (within 1 year after the stroke) (Table 1).

### 3.4 Quality measures of the studies

#### 3.4.1 Internal validity

The quality measures of the studies (Aaronson et al., 2012; Bassetti et al., 2006; Boulos et al., 2015; Broadley et al., 2007; Camilo et al., 2014; Chen et al., 2011; Dziewas et al., 2005; Elkholy et al., 2012; Katzan et al., 2016; Kotzian et al., 2012; Srijithesh et al., 2011) are listed in Table 2. A valid and clinical SDB testing for confirming SDB was required to be included. Seven studies (Aaronson et al., 2012; Bassetti et al., 2006; Camilo et al., 2014; Chen et al., 2011; Elkholy et al., 2012; Katzan et al., 2016; Srijithesh et al., 2011) used full polysomnography and four (Boulos et al., 2015; Broadley et al., 2007; Dziewas et al., 2005; Kotzian et al., 2012) used cardiorespiratory polygraphy as a standard to verify the pre‐screening methods. Two studies (Boulos et al., 2015; Kotzian et al., 2012) succeeded in giving detailed descriptions of the internal quality of the study. The other nine studies (Aaronson et al., 2012; Bassetti et al., 2006; Broadley et al., 2007; Camilo et al., 2014; Chen et al., 2011; Dziewas et al., 2005; Elkholy et al., 2012; Katzan et al., 2016; Srijithesh et al., 2011) failed in giving such information in detail and did not allow a comprehensive evaluation of the risk of bias. More specifically, seven studies (Aaronson et al., 2012; Bassetti et al., 2006; Chen et al., 2011; Dziewas et al., 2005; Elkholy et al., 2012; Katzan et al., 2016; Srijithesh et al., 2011) failed to report the blind execution of index and standard tests. Nine studies (Aaronson et al., 2012; Bassetti et al., 2006; Camilo et al., 2014; Chen et al., 2011; Dziewas et al., 2005; Elkholy et al., 2012; Katzan et al., 2016; Srijithesh et al., 2011) failed to inform whether the index and standard tests were analyzed independently of each other. Finally, eight studies (Aaronson et al., 2012; Bassetti et al., 2006; Broadley et al., 2007; Camilo et al., 2014; Chen et al., 2011; Dziewas et al., 2005; Elkholy et al., 2012; Katzan et al., 2016; Srijithesh et al., 2011) did not report, if the standard test was analyzed independently of clinical information. The evaluation of internal quality in these studies (Aaronson et al., 2012; Bassetti et al., 2006; Broadley et al., 2007; Camilo et al., 2014; Chen et al., 2011; Dziewas et al., 2005; Elkholy et al., 2012; Katzan et al., 2016; Srijithesh et al., 2011) was therefore incomplete.

**Table 2:** The quality measures

Authors, publication year (methods)	Criteria of internal validity					Criteria of external validity					
	Valid reference standard	Apnea definition by standard test	Blind execution of index and standard tests	Prevention of verification bias	Standard test analyzed independently of clinical information	Spectrum of disease	Demographic information validity	Cutoff values for standard test	Missing data reported	Index test completion	Subject selection to standard test
				(Standard and index tests analyzed independently)		(Incl./excl. mentioned) YES/NO	(Age, gender, BMI reported)	(Result for AHI ≥5 or more)			(Patient selection reported)
Questionnaires or prediction model											
Boulos et al. (2015) (4‐Variable, STOP‐BAG, BQ, SOS)	P	Y	Y	Y	Y	Y/Y	Y/Y/Y	Y	Y	Pa	Y
Katzan et al. (2016) (STOP, STOP‐BANG, STOP‐BAG, STOP‐BANG2 STOP‐BAG2 STOP‐BAG2+)	F	Y	U	U	U	N/N	Y/Y/Y	Y	N	Pa	N
Camilo et al. (2014) (BQ, ESS, combined)	F	Y	Y	U	U	Y/Y	Y/Y/Y	Y	N	I	Y
Elkholy et al. (2012) (BQ)	F	Y	U	U	U	Y/Y	Y/Y/Y	Y	N	Pa	N
Kotzian et al. (2012) (BQ)	P	Y	Y	Y	Y	Y/Y	Y/Y/Y	Y	Y	Pa	Y
Srijithesh et al. (2011) (BQ, ESS, combined)	F	Y	U	U	U	Y/Y	Y/N/N	Y	Y	I	Y
Broadley et al. (2007) (MAP index)	P	Y	Y	U	Y	Y/Y	Y/Y/Y	Y	N	U	Y
Bassetti et al. (2006) (ESS, SDQ‐SA)	F	Y	U	U	U	Y/N	Y/Y/Y	Y	Y	Pa	Y
Physiological measures											
Aaronson et al. (2012) (Nocturnal oximetry)	F	Y	U	U	U	Y/Y	Y/Y/Y	Y	Y	Pro	N
Chen et al. (2011) (Nocturia)	F	Y	U	U	U	Y/Y	Y/Y/Y	Y	Y	Pro	Y
Dziewas et al. (2005) (Capnograph)	P	Y	U	U	U	Y/Y	Y/Y/Y	Y	Y	Pro	Y

F, full polysomnograph; I, Informant of the patient; N, no; P, polygraph; Pa, patient; Pro, medical professional, for example nurse; U, unsure; Y, yes.

#### 3.4.2 External validity

The criteria for external validity were fulfilled more thoroughly as all studies succeeded in reporting the cutoff values they used to determine SDB diagnosis against the standard test (Aaronson et al., 2012; Bassetti et al., 2006; Boulos et al., 2015; Broadley et al., 2007; Camilo et al., 2014; Chen et al., 2011; Dziewas et al., 2005; Elkholy et al., 2012; Katzan et al., 2016; Kotzian et al., 2012; Srijithesh et al., 2011). Inclusion and exclusion criteria were provided in nine (Aaronson et al., 2012; Boulos et al., 2015; Broadley et al., 2007; Camilo et al., 2014; Chen et al., 2011; Dziewas et al., 2005; Elkholy et al., 2012; Kotzian et al., 2012; Srijithesh et al., 2011) of the eleven studies. All except one study (Srijithesh et al., 2011) succeeded in reporting basic background information. The missing data were not reported in four studies (Broadley et al., 2007; Camilo et al., 2014; Elkholy et al., 2012; Katzan et al., 2016). One study (Broadley et al., 2007) did not describe the index test completion in detail, and the patient selection method was reported in nine (Bassetti et al., 2006; Boulos et al., 2015; Broadley et al., 2007; Camilo et al., 2014; Chen et al., 2011; Dziewas et al., 2005; Kotzian et al., 2012; Srijithesh et al., 2011) of the eleven studies (Table 2).

### 3.5 Pre‐screening methods for SDB

Eight studies (Boulos et al., 2015; Broadley et al., 2007; Camilo et al., 2014; Elkholy et al., 2012; Katzan et al., 2016; Kotzian et al., 2012; Reitsma et al., 2009; Srijithesh et al., 2011) used sleep questionnaires or a prediction model (Chung, Yegneswaran, Liao, Chung & Vairavanathan, 2008; Douglass, Bornstein, Nino‐Murcia, Keenan & Miles, 1994; Johns, 1991; Maislin, Pack, Kribbs, Smith & Schwartz, 1995; Netzer, Stoohs, Netzer, Clark & Strohl, 1991; Takegami, Hayashino, Chin, Sokejima & Kadotani, 2009), and three physiological measures (Aaronson et al., 2012; Bassetti et al., 2006; Chen et al., 2011) to pre‐screen for SDB or sleep apnea.

#### 3.5.1 Questionnaires or a prediction model

The questionnaires and prediction model included the Berlin Questionnaire (BQ), Epworth Sleepiness Scale (ESS), SOS score, modified Sleep Apnea Scale of the Sleep Disorders Questionnaire (SDQ‐SA), STOP‐BANG and its derivatives, the Four‐variable screening tool (4V) and Multivariate Apnea Index (MAP) (Table 3) (Chung et al., 2008; Douglass et al., 1994; Johns, 1991; Maislin et al., 1995; Netzer et al., 1991; Takegami et al., 2009).

**Table 3:** Diagnostic accuracies of the pre‐screening methods (questionnaires, prediction models and physiological measures) divided into AHI classes

Questionnaires or prediction model								
	Method(s)	AHI cutoff	Sensitivity	Specificity	NPV	PPV	AUC	*p*‐value
AHI ≥ 5/hr								
Katzan et al. (2016)	STOP	AHI ≥ 5/hr	0.80	0.41				
	STOP‐BANG	AHI ≥ 5/hr	0.87	0.56				
	STOP‐BAG	AHI ≥ 5/hr	0.84	0.59				
	STOP‐BANG_2_	AHI ≥ 5/hr	0.87	0.78				
	STOP‐BAG_2_	AHI ≥ 5/hr	0.87	0.78				
	STOP‐BAG_2+_	AHI ≥ 5/hr	0.87	0.80				
Elkholy et al. (2012)	BQ	AHI ≥ 5/hr	0.55	1.00	0.53	1.00		
Srijithesh et al. (2011)	BQ	AHI ≥ 5/hr	0.68	0.59	0.59	0.68		
	BQ & ESS combined	AHI ≥ 5/hr	0.50	0.88	0.58	0.85		
	BQ & ESS separately	AHI ≥ 5/hr	0.77	0.47	0.62	0.65		
AHI ≥ 10–15/hr								
Boulos et al. (2015)	STOP‐BAG	AHI ≥ 10/hr	0.94	0.14	0.48	0.72	0.677	0.012
	4V	AHI ≥ 10/hr	0.97	0.24	0.52	0.90	0.688	0.007
	BQ	AHI ≥ 10/hr	0.53	0.60	0.53	0.60	0.56	0.370
	SOS	AHI ≥ 10/hr	0.53	0.51	0.49	0.56	0.51	0.928
Katzan et al. (2016)	STOP	AHI ≥ 10/hr	0.85	0.40				
	STOP‐BANG	AHI ≥ 10/hr	0.95	0.48				
	STOP‐BAG	AHI ≥ 10/hr	0.91	0.48				
	STOP‐BANG_2_	AHI ≥ 10/hr	0.94	0.53				
	STOP‐BAG_2_	AHI ≥ 10/hr	0.95	0.61				
	STOP‐BAG_2_+	AHI ≥ 10/hr	0.95	0.56				
Camilo et al. (2014)	SOS ‐score	AHI ≥ 10/hr	0.90	0.56	0.96	0.22	0.813	0.005
	BQ	AHI ≥ 10/hr					0.567	0.549
	ESS	AHI ≥ 10/hr					0.789	0.009
Elkholy et al. (2012)	BQ	AHI>15/hr	0.56	0.86	0.63	0.82		
Kotzian et al. (2012)	BQ	AHI ≥ 15/hr	0.69	0.15			0.583	
Srijithesh et al. (2011)	BQ	AHI ≥ 10/hr	0.77	0.54	0.82	0.46		
Broadley et al. (2007)	MAP index >0.5	AHI ≥ 10/hr	0.52	0.81		0.75		
Bassetti et al. (2006)	P‐SA	AHI ≥ 10/hr	0.64	0.67				
AHI ≥ 20/hr								
Katzan et al. (2016)	STOP	AHI ≥ 20/hr	0.89	0.33				
	STOP‐BANG	AHI ≥ 20/hr	0.82	0.54				
	STOP‐BAG	AHI ≥ 20/hr	0.76	0.58				
	STOP‐BANG_2_	AHI ≥ 20/hr	0.82	0.63				
	STOP‐BAG_2_	AHI ≥ 20/hr	0.82	0.62				
	STOP‐BAG_2+_	AHI ≥ 20/hr	0.82	0.70				
AHI ≥ 30/hr								
Katzan et al. (2016)	STOP	AHI>30/hr	0.88	0.30				
	STOP‐BANG	AHI>30/hr	0.83	0.49				
	STOP‐BAG	AHI>30/hr	0.76	0.53				
	STOP‐BANG_2_	AHI>30/hr	0.83	0.59				
	STOP‐BAG_2_	AHI>30/hr	0.83	0.53				
	STOP‐BAG_2+_	AHI>30/hr	0.83	0.56				
Camilo et al. (2014)	SOS score	AHI ≥ 30/hr	0.90	0.30	0.97	0.92	0.686	0.048
	BQ	AHI ≥ 30/hr					0.622	0.191
	ESS	AHI ≥ 30/hr					0.646	0.119
Elkholy et al. (2012)	BQ	AHI>30/hr	0.67	0.83	0.79	0.72		
Physiological measures								
Aaronson et al. (2012)	Nocturnal Oximetry (Index ≥ 15/hr)	AHI ≥ 15/hr	0.77	1.00	0.83	1.00	0.93	
Chen et al. (2011)	Nocturia	AHI ≥ 30/hr	0.68	0.59			0.70	
Dziewas et al. (2005)	Capnograph (Index >5/hr)	AHI > 10/hr r	0.87	1.00	0.86	1.00		

AHI, Apnea‐Hypopnea Index; AUC, Area Under Curve; BQ, Berlin Questionnaire; ESS, Epworth Sleepiness Scale; NPV, Negative Predictive Value; PPV, Positive Predictive Value; P‐SA, Probable Sleep Apnea (defined by ESS >10 or SDQ‐SA ≥ 32 in women and ≥36 in men); SDQ‐SA, Sleep Disorders Questionnaire.

Berlin Questionnaire (Netzer et al., 1991) is a questionnaire used in sleep apnea diagnostics in primary care settings. It evaluates the risk for sleep apnea. It was used in five studies (Boulos et al., 2015; Camilo et al., 2014; Elkholy et al., 2012; Kotzian et al., 2012; Srijithesh et al., 2011).

Epworth Sleepiness Scale (Johns, 1991) assesses daytime sleepiness by evaluating the tendency to fall asleep in given situations. ESS was used in two studies (Camilo et al., 2014; Srijithesh et al., 2011). *SOS score* (Camilo et al., 2014) combines the elements of BQ (Netzer et al., 1991) and ESS (Johns, 1991) by adding both together with a modified scoring. It was demonstrated in two studies (Boulos et al., 2015; Camilo et al., 2014).

Sleep Disorders Questionnaire (*SDQ‐SA*) (Douglass et al., 1994) is a basic sleep apnea questionnaire used commonly in SDB pre‐screening in clinical research. One study (Bassetti et al., 2006) used SDQ‐SA (Douglass et al., 1994) in combination with ESS (Johns, 1991) to predict SDB so that Probable Sleep Apnea (P‐SA) was defined by ESS > 10 or SDQ‐SA ≥ 32 in women and ≥36 in men.

STOP‐BANG (Chung et al., 2008) is a questionnaire validated to measure SDB by asking about snoring, tiredness, observed pauses in breathing during sleep, blood pressure, BMI, age, neck circumference, and gender. Two studies (Boulos et al., 2015; Katzan et al., 2016) used *STOP‐BAG*, which is otherwise the same as STOP‐BANG but without the neck circumference measurement. Additionally, one study (Katzan et al., 2016) used and compared various models derived from STOP‐BANG such as *STOP, STOP‐BANG2, STOP‐BAG2,* and *STOP‐BAG2+,* where the BANG part is replaced with modified scoring consisting of continuous variables and additional factors.

Four‐variable screening tool (Takegami et al., 2009) is a sleep questionnaire validated to assess moderate to severe SDB with variables consisting of sex, BMI, blood pressure, and snoring. The 4V screening tool was assessed in one study (Boulos et al., 2015).

Multivariate Apnea Index index (Maislin et al., 1995) has been used in predicting obstructive sleep apnea. It uses the patient's age, sex, and BMI in the prediction model. *MAP* was used in sleep apnea evaluation in one study (Broadley et al., 2007).

#### 3.5.2 Physiological measures

Three studies (Aaronson et al., 2012; Chen et al., 2011; Dziewas et al., 2005) used physiological methods for SDB pre‐screening and compared the results with a standard sleep apnea test. One study (Aaronson et al., 2012) measured neurologic patients overnight with *a common oximetry,* one (Chen et al., 2011) tested *the frequency of void* between bedtime and waking time, and one (Dziewas et al., 2005) measured *the expired CO2 levels* from which apneas and hypopneas were identified.

### 3.6 The accuracy and predictive methods

The results of the diagnostic accuracies (Chung et al., 2008; Douglass et al., 1994; Johns, 1991; Maislin et al., 1995; Netzer et al., 1991; Takegami et al., 2009) are listed in Table 3.

### 3.7 Questionnaires or prediction model

The definitions of cutoff values for AHI between the studies were highly non‐uniform. For example, some studies set the cutoff for AHI to 10 and some to 15. Therefore, we divided the studies into four groups according to the AHI cutoffs the authors had used as follows: AHI ≥ 5/hr, AHI ≥ 10–15/hr, AHI ≥ 20, AHI ≥ 30 (Table 3).

Three studies (Elkholy et al., 2012; Katzan et al., 2016; Srijithesh et al., 2011) reported the results with a cutoff of AHI ≥ 5/hr. One study (Srijithesh et al., 2011) tested BQ, BQ and ESS separately and in combination but none of them showed particularly high specificities or sensitivities in predicting SDB. One study (Elkholy et al., 2012) also assessed BQ and found it to be highly specific but not highly sensitive in predicting SDB. STOP‐BANG_2_, STOP‐BAG_2,_ and STOP‐BAG_2+_ showed moderate sensitivity and specificity to SDB with 5/hr ≤ AHI<10/hr cutoff (Table 3) (Katzan et al., 2016).

All studies that used questionnaires or prediction models reported the results with AHI cutoff of either 10 or 15 (AHI ≥ 10–15/hr) (Bassetti et al., 2006; Boulos et al., 2015; Broadley et al., 2007; Camilo et al., 2014; Elkholy et al., 2012; Katzan et al., 2016; Kotzian et al., 2012; Srijithesh et al., 2011). The sensitivities ranged from 52.0% to 100% and specificities from 14.0% to 100%. The most sensitive instrument in predicting SDB was the 4V questionnaire with 97.0% sensitivity. The specificity, however, was only 24% and the area under curve (AUC) was poor, that is, 67.7%. Hence, this instrument recognizes the affected ones well but those non‐affected poorly. The most specific instrument in the moderate SDB group was BQ with 86% specificity. Sensitivity, however, was only 56% indicating that nearly half of the affected ones remain unidentified. One study used AHI cutoff point ≥20/hr (Katzan et al., 2016) (Table 3).

The same trend continued in the category with the highest cutoff for AHI (AHI ≥ 30/hr) as SOS‐score, BQ, ESS, and STOP‐BANG, and its derivatives indicated good sensitivities to predict SDB but low specificities (Table 3) (Camilo et al., 2014; Elkholy et al., 2012; Katzan et al., 2016).

### 3.8 Physiological measures

Three studies (Aaronson et al., 2012; Chen et al., 2011; Dziewas et al., 2005) used physiological measures (capnography, nocturnal oximetry, nocturia) to test their power to predict SDB (Table 3). The most sensitive and specific of these was the capnography (Dziewas et al., 2005) measurement with sensitivity, specificity, and negative and positive predictive values of 87.0%, 100%, 86.0%, and 100%, respectively. The corresponding values of the nocturnal oximetry (Aaronson et al., 2012) were 77.0%, 100%, 83.0%, and 100%, and nocturia (Chen et al., 2011) 68.0% and 59.0% with no reported NPV and PPV. There were no reports on the demands for resources such as personnel or time of physiological measures compared to standard tests for SDB (polysomnography or cardiorespiratory polygraphy).

### 3.9 Further quantitative analyses

Further quantitative analyses or a meta‐analysis was not carried out due to the heterogeneity within the reported methods and measures (AHI cutoff points) of the studies (Aaronson et al., 2012; Bassetti et al., 2006; Boulos et al., 2015; Broadley et al., 2007; Camilo et al., 2014; Chen et al., 2011; Dziewas et al., 2005; Elkholy et al., 2012; Katzan et al., 2016; Kotzian et al., 2012; Srijithesh et al., 2011).

## 4 DISCUSSION

To our knowledge, this is the first systematic review on SDB pre‐screening methods in acute and subacute stroke patients. Pre‐screening methods for detecting SDB after stroke have not been studied extensively, as only eleven different pre‐screening methods for Sleep‐Disordered Breathing after acute and subacute stroke were identified. The results show that some pre‐screening methods might have the potential to identify patients suffering from SDB before polygraphy for targeted testing. Questionnaires are more desirable pre‐screening methods due to their simplicity as they can be self‐answered or filled in by a nurse on the basis of an interview at a stroke unit. Even if the questionnaires are easy and fast to administer, their predictive value was proved to be poor, and they cannot be clinically recommended for SDB screening after stroke. The physiological measures (capnography, nocturnal oximetry) produced the best predictive results but their usability for screening is greatly diminished due to their resource needs, that is, equipment, time‐consuming overnight monitoring, and specialist interpretation on data.

The overall quality of the included studies (Aaronson et al., 2012; Bassetti et al., 2006; Boulos et al., 2015; Broadley et al., 2007; Camilo et al., 2014; Chen et al., 2011; Dziewas et al., 2005; Elkholy et al., 2012; Katzan et al., 2016; Kotzian et al., 2012; Srijithesh et al., 2011) was modest as assessed according to the Cochrane Methods Group guidelines (Reitsma et al., 2009). Only two studies (Boulos et al., 2015; Kotzian et al., 2012) were methodologically good while nine other studies (Aaronson et al., 2012; Bassetti et al., 2006; Broadley et al., 2007; Camilo et al., 2014; Chen et al., 2011; Dziewas et al., 2005; Elkholy et al., 2012; Katzan et al., 2016; Srijithesh et al., 2011) showed quality issues. The cohorts in the studies selected for the systematic review (Aaronson et al., 2012; Bassetti et al., 2006; Boulos et al., 2015; Broadley et al., 2007; Camilo et al., 2014; Chen et al., 2011; Dziewas et al., 2005; Elkholy et al., 2012; Katzan et al., 2016; Kotzian et al., 2012; Srijithesh et al., 2011) were homogeneous since the inclusion and exclusion criteria were similar in all. For example, all the studies tested only patients in stable medical conditions as none of the studies included comatose patients, because pre‐screening methods requiring co‐operation cannot be done in such patients, though these patients are probably in the most severe risk of having altered breathing patterns during sleep. Moreover, the studies excluded patients with diseases altering sleep or breathing such as depression or chronic obstructive pulmonary disease. Also, only patients with first ever stroke were included leaving out all the patients with recurrent strokes.

There were some limitations regarding the studies selected in the present systematic review (Aaronson et al., 2012; Bassetti et al., 2006; Boulos et al., 2015; Broadley et al., 2007; Camilo et al., 2014; Chen et al., 2011; Dziewas et al., 2005; Elkholy et al., 2012; Katzan et al., 2016; Kotzian et al., 2012; Srijithesh et al., 2011). It was difficult to compare the results due to the heterogeneity of the reporting methods or AHI cutoff values in moderate SDB within the studies. Therefore, we needed to divide the AHI groups into four classes which may not be clinically relevant as AHI cutoff value 15 is usually used in clinical situations, when judging the need for positive airway pressure device while 30 indicates critical SDB requiring an immediate positive airway pressure device. For example, SDB severity cutoffs used in analyses varied between the studies making the sample too heterogeneous for an overall pooled analysis or meta‐analysis. There was variation in the patient cohorts and in measures, that is, in the number of participants, duration of stroke, stroke severity, and measures of SDB within as well as between studies. For example, in one study (Katzan et al., 2016) the time between the stroke and sleep testing varied considerably between the patients. Also, most of the studies lacked some information of internal and external validity details thus decreasing the overall quality of the studies.

Extensive research has emphasized that SDB is highly prevalent after stroke and there might even be a causal relationship between SDB and stroke (Bassetti et al., 1996; Cam et al., 2013; Gao et al., 2010; Hermann & Bassetti, 2009; Martínez‐García et al., 2009; Ou et al., 2015; Sahlin et al., 2008; Yaggi et al., 2005). In fact, sleep apnea is generally recognized as an independent risk factor for stroke (Yaggi et al., 2005). The problem is in identifying those at risk because the resources to screen for SDB are limited in neurology departments due to the primary duties the health care workers have in securing the patients’ conditions. A clinical instrument, easy for the hospital personnel to administer for targeted SDB pre‐screening and accurate in differentiating between SDB positive and SDB negative patients, is needed to reduce the unnecessary measurements and keep the extra work at minimum for efficient use of the clinician's time.

For pre‐screening SDB in acute and subacute stroke patients, questionnaires can be conducted quickly and the results can be assessed immediately. However, the existing literature does not fully succeed in reassuring the functionality of questionnaires as a SDB pre‐screening method, because the diagnostic accuracies were altogether modest. For example, although performing well in identifying the SDB positive patients, only every fourth patient would be accurately diagnosed as non‐affected with a 4V questionnaire due to the low specificity of the test resulting in a high number of false positives. This is not useful in decreasing the work of specialized physicians and therefore not a very practical pre‐screening instrument. Moreover, there was some discrepancy in the results of the Berlin Questionnaire (BQ) (Netzer et al., 1991), as they varied tremendously between studies which affect detrimentally the creditability of its use in SDB screening in neurologic patients. For example, the majority of studies using BQ (Boulos et al., 2015; Camilo et al., 2014; Kotzian et al., 2012; Srijithesh et al., 2011) concluded that BQ is not sufficient in predicting SDB at the moderate severity level, while one study (Elkholy et al., 2012) recommended the opposite. Also, when looking at the specificities between these studies, it is impossible not to question the plausibility of the results as they vary considerably (Table 3). The heterogeneity of the methods and different cutoff points for moderate AHI prevented us from performing a meta‐analysis on the pre‐screening methods for SDB after stroke.

To conclude, no plausible and pragmatic tool for clinical pre‐testing of SDB exists according to our systematic review. Currently, no specific SDB pre‐screening method can be referred to clinicians working in neurologic departments. Thus, polysomnography or cardiorespiratory polygraphy is still clinically needed when suspecting SDB in stroke patients. Still, the high SDB prevalence among stroke patients remains and the physicians in stroke units need to discretionarily prescribe further sleep testing to susceptible patients. More research is needed to find an optimal pre‐screening instrument for clinical practice to identify SDB patients after stroke.

## CONFLICT OF INTEREST

Mrs. M. Takala reports no disclosures relevant to the manuscript. Adjunct professor Puustinen reports personal fees from consultancy of translation proof‐reading (PharmaQuest Ltd), lecturing fees (Finnish Neurological Association, Kankaanpää Institute, Society for Memory Disorders Expertise in Finland, Multiple Sclerosis Association of Trauma Region, Boehringer‐Ingelheim, Orion, and Sanofi‐Aventis), preparing manuscripts (Journal of Finnish Medical Association, Journal of General Practitioners in Finland), and personal fees from travel, accommodation, and conference expenses (Abbvie, Roche), outside the submitted work. Adjunct professor Rauhala reports no disclosure relevant to the manuscript. Adjunct professor Holm reports personal fees from travel and accommodation expenses (Resmed), outside the submitted work.
