# Supplementary material for: Pre‐screening of sleep‐disordered breathing after stroke: A systematic review
Source: Brain Behav. 2018 Oct 29;8(12):e01146. doi: 10.1002/brb3.1146 (PMC6305929; doi:10.1002/brb3.1146)
Supplement: Supplementary file 1 [file BRB3-8-e01146-s001.docx]

**SUPPLEMENTARY MATERIAL**

**Supplementary Table.** The excluded studies and reasons for exclusion

| **Authors, year** | **Summary** | **Reason for exclusion** |
| --- | --- | --- |
| Severine et al. 2016^S1^ | The study assessed the power of STOP-BANG questionnaire in SDB screening in acute cerebrovascular stroke patients. | The study did not assess the diagnostic accuracy of index test against standard test. |
| Chen et al. 2015a^S2^ | The study assessed the relationship between SDB and biomarkers after cerebrovascular stroke. | As above. |
| Chen et al. 2015b^S3^ | The study investigated whether OSA independently increases arterial stiffness in ischemic stroke patients using carotid emoral pulse wave velocity and the central augmentation index. | As above. |
| Chernysev et al. 2015^S4^ | The study assessed the validity of portable out-of-center cardiopulmonary sleep testing in confirming SDB diagnosis against full polysomnography. | The index test in the study does not fulfill the idea of a pre-screening method |
| Gupta et al. 2015a^S5^ | The study assessed the prevalence of restless legs syndrome among patients with cerebrovascular stroke. Also the study explored the anatomical correlation between the location of stroke and restless legs syndrome. | The study did not assess the diagnostic accuracy of index test against standard test. |
| Klobucnikova et al. 2015^S6^ | The study assessed the prevalence of SDB and daytime sleepiness in acute cerebrovascular stroke patients. Also, the association of SDB, stroke severity, and location of stroke with EDS was analyzed. | As above. |
| Väyrynen et al. 2014^S7^ | The study assessed the feasibility of Emfit sleep mattress in SDB screening in acute cerebrovascular stroke patients. | The study failed to use valid standard test to compare the index test with. |
| Kepplinger et al. 2013^S8^ | The study assessed the feasibility of early sleep apnea screening in stroke unit, and its effect on post-discharge sleep apnea care. Also, the association of sleep apnea and clinical features was examined. | The study did not assess the diagnostic accuracy of index test against standard test. |
| Medeiros et al. 2012^S9^ | The study assessed the association between respiratory abnormalities and atherogenic pro-inflammatory markers. | As above. |
| Aaronson et al. 2012^S10^ | The study assessed the predictive power of self-reported symptoms, sociodemographic and clinical parameters in SDB recognition after cerebrovascular stroke. | The study failed to use valid standard test to compare the index test with. |
| Kunz et al. 2012^S11^ | The study assessed the association of increased plasma concentration of biomarkers with SDB in patients after acute cerebrovascular stroke. | As above. |
| Jiang et al. 2011^S12^ | The study assessed the subjective sleep quality and polysomnographic sleep structure in patients with stroke. | The study did not assess the diagnostic accuracy of index test against standard test. |
| Joo et al. 2011^S13^ | The study assessed the prevalence of SDB and variables predicting it after cerebrovascular stroke. | As above. |
| Mansukhani et al. 2011^S14^ | The study evaluated the prevalence and risk of OSA in acute cerebrovascular stroke patients. The relationship between OSA and age, sex, stroke subtype, disability and death was examined as well. | As above. |
| Medeiros et al. 2011^S15^ | The study assessed the prevalence of restless legs syndrome after acute cerebrovascular stroke, and its relationship with sleep disturbance and clinical outcome during long-term follow-up. | As above. |
| Arzt et al. 2010^S16^ | The study compared the degree of excessive daytime sleepiness and BMI between different SDB severity groups. | As above. |
| Brooks et al. 2010^S17^ | The study assessed the prevalence of SDB after cerebrovascular stroke and the relationship of respiratory index and health survey variables. | As above. |
| Chan et al. 2010^S18^ | The study assessed the prevalence of SDB after cerebrovascular stroke. Additionally, the relationship between respiratory disturbance and clinical features was assessed. | As above. |
| Kaneko et al. 2003^S19^ | The study assessed the association of sleep apnea and greater degree of functional disability and longer hospitalization among cerebrovascular stroke patients | As above. |
| Harbison et al. 2002^S20^ | The study assessed the prevalence and course of SDB after acute cerebrovascular stroke. Additionally, the association of SDB with age, stroke subtype, pre-stroke functionality and post-stroke outcome was examined. | As above. |
| Hui et al. 2002^S21^ | The study assessed the prevalence of SDB, CPAP adherence and acceptance in ischemic stroke patients. | As above. |
| Wessendorf et al. 2002^S22^ | The study assessed the use of nocturnal oximetry in SDB screening in acute cerebrovascular stroke patients. The diagnostic accuracy was examined. | No English full-text of the study was found. |
| Iranzo et al. 2002^S23^ | The study assessed the prevalence of sleep apnea, clinical presentation, course and functional outcome in ischemic stroke patients. | The study did not assess the diagnostic accuracy of index test against standard test. |
| Wessendorf et al. 2000^S24^ | The study assessed the prevalence of SDB in patients with first-ever cerebrovascular stroke. The types of SDB were assessed and the association of SDB with known risk factors of stroke. | As above. |
| Bassetti et al. 1997^S25^ | The study assessed the prevalence of SDB after cerebrovascular stroke.  Additionally, the predictive factors were assessed. | As above. |
| Good et al. 1996^S26^ | The study assessed the association of arterial oxyhemoglobin desaturation in recent ischemic stroke patients and functional outcome. | As above. |

**References**

S1. Severine JE, Thanavaro J, Lorenz R et al. Screening for Obstructive Sleep Apnea in Hospitalized Transient Ischemic Attack Stroke Patients Using the STOP-Bang Questionnaire. *The Journal for Nurse Practitioners*. 2016:12(1):19-26.

S2. Chen CY, Chen CL, Yu CC. Obstructive sleep apnea is independently associated with arterial stiffness in ischemic stroke patients. *J Neurol.* 2015:262(5):1247-1254.

S3. Chen X, Bi H, Zhang M, Liu H, et al. Research of Sleep Disorders in Patients with Acute Cerebral Infarction. *J Stroke Cerebrovasc Dis.* 2015:24(11):2508-2513.

S4. Chernyshev OY, McCarty OY, Moul DE, et al. A pilot study: portable out-of-center sleep testing as an early sleep apnea screening tool in acute ischemic stroke. *Nat Sci of Sleep.* 2015:7:127-138.

S5. Gupta A, Shukla G, Mohammed A et al. Restless legs syndrome, a predictor of subcortical stroke: a prospective study in 346 stroke patients. *Sleep Med.* 2017:29:61-67. doi: 10.1016/j.sleep.2015.05.025. Epub 2015 Jul 20.

S6. Klobucnikova K, Siarnik P, Carnicka et al. Causes of Excessive Daytime Sleepiness in Patients with Acute Stroke-A Polysomnographic Study. *J Stroke Cerebrovasc Dis*. 2016:25(1):83-6. doi: 10.1016/j.jstrokecerebrovasdis.2015.08.038. Epub 2015 Sep 26

S7. Väyrynen K, Kortelainen K, Numminen H et al. Screening sleep disordered breathing in stroke unit. *Sleep Disord*. 2014:317615. doi: 10.1155/2014/317615.

S8. Kepplinger J, Barlinn K, Albright KC et al. Early sleep apnea screening on a stroke unit is feasible in patients with acute cerebral ischemia. *J Neurol.*2013:260(5):1343-1350.

S9. Medeiros CA, de Bruin VM, Andrade GM et al. Obstructive sleep apnea and biomarkers of inflammation in ischemic stroke. *Acta Neurol Scand.* 2012:126(1):17-22.

S10. Aaronson JA, van Bezeij T, van den Aardweg JG et al. Diagnostic accuracy of nocturnal oximetry for detection of sleep apnea syndrome in stroke rehabilitation. *Stroke*. 2012:43(9):2491-2493.

S11. Kunz AB, Kraus J, Young P et al. Biomarkers of inflammation and endothelial dysfunction in stroke with and without sleep apnea. *Cerebrovasc Dis.* 2012:33(5):453-460.

S12. Jiang L, Wu X. [Research on the relationship between sleep phases and heart rate variability]. *Sheng Wu Yi Xue Gong Cheng Xue Za Zhi.* 2011:28(1):148-152.

S13. Joo BE, Seok HY, Yu SW et al. Prevalence of sleep-disordered breathing in acute ischemic stroke as determined using a portable sleep apnea moni-toring device in Korean subjects. *Sleep Breath.* 2011:15(1):77-82.

S14. Mansukhani MP, Bellolio MF, Kolla BP et al. Worse outcome after stroke in patients with obstructive sleep apnea: an observational cohort study. *J Stroke Cerebrovasc Dis*. 2011:20(5):401-405.

S15. Medeiros CA, de Bruin PF, Paiva TR et al. Clinical outcome after acute ischaemic stroke: the influence of restless legs syndrome. *Eur J Neurology.* 2011:18(1):144-149. doi:10.1111/j.1468-1331.2010.03099.x

S16. Arzt M, Young T, Peppard PE et al. Dissociation of obstructive sleep apnea from hypersomnolence and obesity in patients with stroke. *Stroke*. 2010:41(3):e129-134.

S17. Brooks D, Davis L, Vujovic-Zotovic N et al. Sleep-disordered breathing in patients enrolled in an inpatient stroke rehabilitation program. *Arch Phys Med Rehabil*. 2010:91(4):659-662.

S18. Chan W, Coutts SB, Hanly, P. Sleep apnea in patients with transient ischemic attack and minor stroke: opportunity for risk reduction of recurrent stroke? *Stroke.* 2010:41(12):2973-2975.

S19. Kaneko Y, Hajek VE, Zivanovic V et al. Relationship of sleep apnea to functional capacity and length of hospitalization following stroke. *Sleep*. 2003:26(3):293-297.

S20. Harbison J, Ford GA, James OF et al. Sleep-disordered breathing following acute stroke. *Qjm.* 2002:95(11):741-747.

S21. Hui DS, Choy DK, Wong LK et al. Prevalence of sleep-disordered breathing and continuous positive airway pressure compliance: results in Chinese patients with first-ever ischemic stroke. *Chest.* 2002:122(3):852-860.

S22. Wessendorf TE, Alymov G, Wang YM et al. [Pulse oximetry screening for sleep-disordered breathing in stroke]. *Pneumologie*. 2002:56(6):357-362.

S23. Iranzo A, Santamaria J, Berenguer J et al. Prevalence and clinical importance of sleep apnea in the first night after cerebral infarction. *Neurology*. 2002:58(6):911-916.

S24. Wessendorf TE, Teschler H, Wang YM et al. Sleep-disordered breathing among patients with first-ever stroke. *J Neurol.* 2002:247(1):41-47.

S25. Bassetti C, Aldrich MS, Quint, D. Sleep-disordered breathing in patients with acute supra- and infratentorial strokes. A prospective study of 39 patients. *Stroke.* 1997:28(9):1765-1772.

S26. Good DC, Henkle JQ, Gelber D et al. Sleep-disordered breathing and poor functional outcome after stroke. *Stroke*. 1996:27(2):252-259.
